# Hybrid glasses from strong and fragile metal-organic framework liquids

**DOI:** 10.1038/ncomms9079

**Published:** 2015-08-28

**Authors:** Thomas D. Bennett, Jin-Chong Tan, Yuanzheng Yue, Emma Baxter, Caterina Ducati, Nick J. Terrill, Hamish H. -M. Yeung, Zhongfu Zhou, Wenlin Chen, Sebastian Henke, Anthony K. Cheetham, G. Neville Greaves

**Affiliations:** 1 Department of Materials Science and Metallurgy, University of Cambridge, Charles Babbage Road, Cambridge CB3 0FS, UK; 2 Department of Engineering Science, University of Oxford, Parks Road, Oxford OX1 3PJ, UK; 3 Section of Chemistry, Aalborg University, DK-9220 Aalborg, Denmark; 4 State Key Laboratory of Silicate Materials for Architectures, Wuhan University of Technology, Wuhan 430070, China; 5 Diamond Light Source Ltd, Diamond House, Harwell Science and Innovation Campus, Didcot OX11 0DE, UK; 6 International Center of Materials Nanoarchitectonics (MANA), National Institute for Materials Science (NIMS), Namiki 1-1, Tsukuba, Ibaraki 305-0044, Japan; 7 Institute of Mathematics, Physics and Computer Science, Department of Physics, Aberystwyth University, Aberystwyth SY23 3BZ, UK; 8 Lehrstuhl für Anorganische Chemie II, Fakultät für Chemie und Biochemie, Ruhr-Universität Bochum, Universitätsstraße 150, 44801 Bochum, Germany

## Abstract

Hybrid glasses connect the emerging field of metal-organic frameworks (MOFs) with the glass formation, amorphization and melting processes of these chemically versatile systems. Though inorganic zeolites collapse around the glass transition and melt at higher temperatures, the relationship between amorphization and melting has so far not been investigated. Here we show how heating MOFs of zeolitic topology first results in a low density ‘perfect' glass, similar to those formed in ice, silicon and disaccharides. This order–order transition leads to a super-strong liquid of low fragility that dynamically controls collapse, before a subsequent order–disorder transition, which creates a more fragile high-density liquid. After crystallization to a dense phase, which can be remelted, subsequent quenching results in a bulk glass, virtually identical to the high-density phase. We provide evidence that the wide-ranging melting temperatures of zeolitic MOFs are related to their network topologies and opens up the possibility of ‘melt-casting' MOF glasses.

## Abstract (web summary)

The fine interface between crystallinity and amorphicity in synthetic hybrid materials has to-date been relatively under-explored. Here, the authors probe the relationship between amorphisation and melting behaviour in zeolitic metal-organic frameworks as a route towards functional glasses.

## Graphical abstract

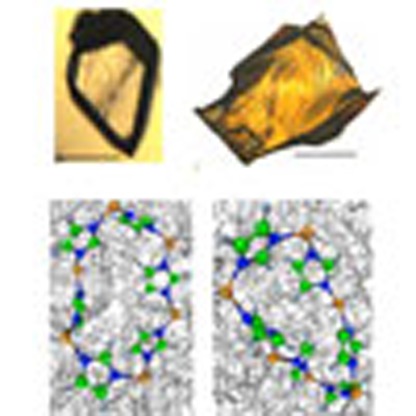

The microporous hybrid materials known as metal-organic frameworks (MOFs) consist of inorganic clusters or ions bridged by organic ligands in open, as well as dense three-dimensional arrays. The former are of great interest owing to their potential use in gas separation and storage, and the latter in multiferroic, conductive and drug/harmful waste encapsulation applications^1^,^2^,^3^. An important subset of MOFs, the zeolitic imidazolate frameworks (ZIFs), adopt similar network structures to zeolites (inorganic low-density frameworks of corner sharing SiO_4_ and AlO_4_ tetrahedra), and, in particular, undergo thermal and pressure-induced amorphization (loss of periodicity)^4^,^5^,^6^,^7^. High-density amorphous (HDA) inorganic glasses, along with low-density amorphous (LDA) states, of identical topologies to their parent crystalline phases have previously been identified via zeolite amorphization^4^,^6^.

Such LDA states are also referred to as ‘perfect' glasses^8^, and were first observed by depressurizing pressure-induced HDA phases or desolvating crystalline structures in, for example, ice^9^, silicon^10^ and trehalose^11^. LDA phases are of scientific interest because of their location deep in the potential energy landscape (PEL)^12^,^13^, at similar potential energies to their crystalline equivalents. This is in contrast to HDA phases that share the same composition as their perfect glass LDA counterparts, have greater entropy as well as density, and are located higher in the PEL. Moreover, compared with HDA phases, LDA phases have unique mechanical properties^12^,^14^, which are connected to the formation of ultrastable glasses^15^. In particular, perfect glasses have been predicted to soften to super-strong liquids (low-density liquid (LDLs))^8^ above the glass transition temperature *T*_g_ in the supercooled state.

Experimentally, for most glass systems *T*_g_∼2/3 *T*_m_, defining the practical limits of the supercooled state^12^. For glasses with a well-defined *T*_g_, but that happen to decompose on heating before they melt, this relationship offers the opportunity to project a ‘virtual melting point' that can be compared with the actual melting points *T*_m_ of isomorphous systems that survive the transition at *T*_m_ from the supercooled to the liquid state.

The dynamic behaviour of a supercooled liquid is quantified through the fragility index, *m*, equation (1), which measures, on a reduced temperature scale, the activation energy of the viscosity *η* at the glass transition *T*_g_ (ref. 16). *T*_g_ is defined to occur when *η* reaches 10^12^ Pa.s. While silica is the strongest liquid among conventional glass-forming liquids, fragilities for some LDL phases fall between 12 (ref. 13) and 14 (ref. 17), endorsing them as super-strong liquids, the antecedents of perfect glasses^7^,^12^.


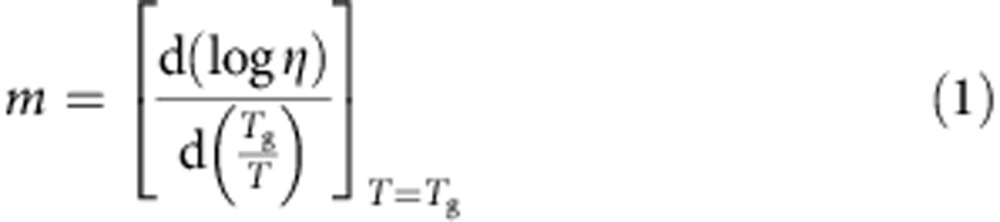


In the context of forming glasses from the collapse of zeolitic structures, the prospect was that this might lead to glasses sharing similar topology to precursor crystals^12^. Indeed the ordered nature of the LDA perfect (low entropy) glass phase was identified by the retention of the THz features defining zeolitic topology when the majority of the starting crystal had amorphized^6^. This was in contrast to the eventual emergence of a featureless boson peak at higher temperatures, typical of less well-ordered conventional higher entropy HDA glasses^12^. Glass transition temperatures of LDA phases were found to be significantly greater than HDA phases with fragilities in the super-strong range^4^,^13^. Finally, by combining temperature- and pressure (*P*)-induced amorphization experiments, a critical point was identified at negative pressure^13^ and a negative d*T*/d*P* slope for the LDL–high-density liquid (HDL) transition. Considering the Clapeyron relation, d*T*/d*P*=Δ*V*/Δ*S*, if d*T*/d*P* is negative, an increase in entropy (*S*) signifies a decrease in volume (*V*) and increase in density, endorsing the LDA and HDA assignments that had been made.

In the present work, we turn our attention to MOFs, research of the glassy behaviour of which is scarce^18^,^19^. Specifically, we contrast ZIF-4 [Zn(C_3_H_3_N_2_)_2_] with ZIF-8 [Zn(C_4_H_5_N_2_)_2_] (ref. 20). We study the mechanism of amorphization of ZIF-4 by thermogravimetric analysis, differential scanning calorimetry (DSC), X-ray total scattering and *in situ* small- and wide-angle X-ray scattering (small angle X-ray scattering (SAXS)/wide angle X-ray scattering (WAXS)) experiments. Importantly, the different *T*_g_s of the LDA, HDA and melt-quenched hybrid glass (MQG) reflect their differing depths in the PEL and the differences in fragility of the corresponding supercooled liquids. While ZIF-8 decomposes before it melts, the ‘virtual' *T*_m_ discussed above can be calculated, lying close to the ‘real' *T*_m_ of its inorganic counterpart. This suggests the dominance of network architecture in melting, characterized by collective THz vibrations^6^, in contrast to the simplistic interatomic variance condition for melting enshrined in Lindemann's law^21^.

## Results

### Differential scanning calorimetry

**Figure 1:**
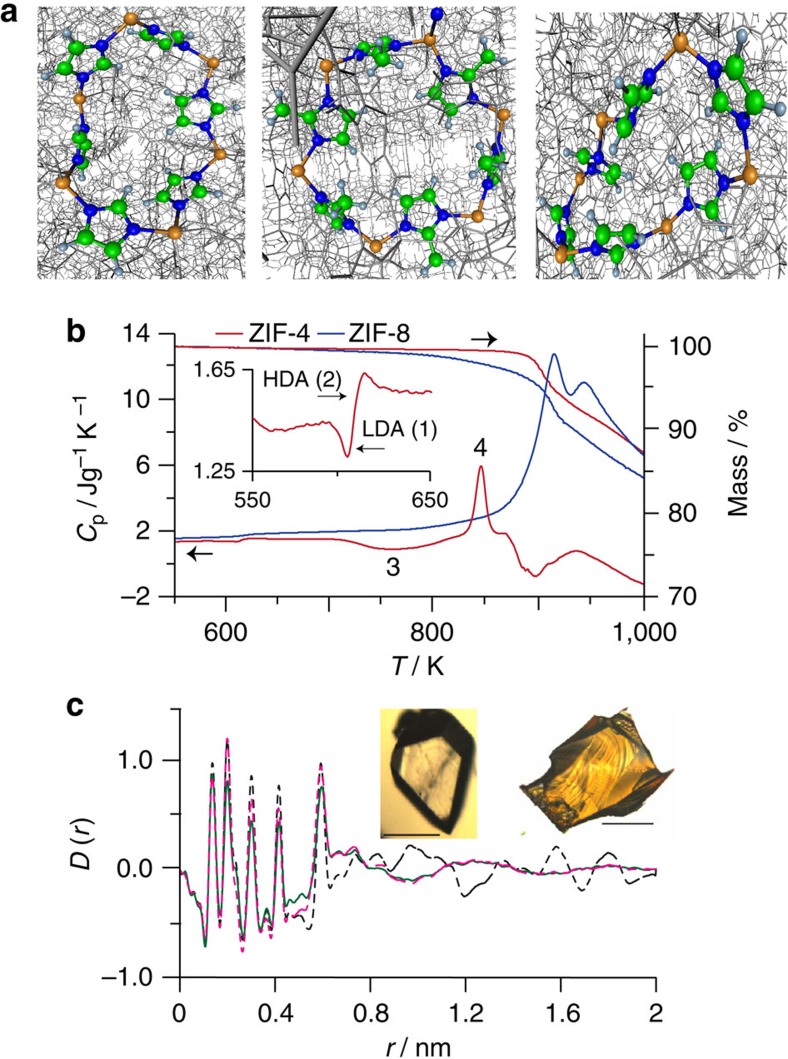
Phase transitions of ZIF-4 on heating. (**a**) Highlighting the rings and imidazolate linkages in zeolitic topologies, in the ordered structure of crystalline ZIF-4 (left) and ZIF-8 (center), and the disordered HDA phase (right) obtained by Molecular Dynamics modelling (Supplementary Methods). Zn, orange; N, blue; C, green; and H, grey. (**b**) Thermogravimetric analysis and *C*_p_ plots for ZIF-4 and ZIF-8, showing, for the former (inset), exothermic collapse to the LDA phase (1) which is closely followed by (2) endothermic formation of the HDA phase, and (3) recrystallization (exothermic). Endothermic melting (4) then follows before thermal degradation. (**c**) X-ray PDF data *D(r)* measured for the MQG (green), ZIF-4 (broken black) and the HDA phase (broken pink). The X-ray total scattering data *S*(*q*) is presented in Supplementary Fig. 1. Inset: optical images of (left) ZIF-4 (right) MQG, showing the typical fracture pattern of a non-metallic bulk glass. Scale bars, 100 μm.

ZIF-4 collapses to an HDA phase, through formation of a LDA phase, before recrystallization into the dense ZIF-zni structure, which can be remelted. Quenching from below the decomposition temperature leads to the formation of a bulk MQG, virtually indistinguishable from the HDA phase, despite each having totally different thermal histories (Fig. 1). *T*_m_ for ZIF-4 lies close to that of an inorganic phosphate with a related zeolitic topology^22^. ZIF-8, on the other hand, adopts the sodalite structure, and does not thermally collapse, though amorphizes under pressure^23^.

### Small and wide angle X-ray scattering

**Figure 2:**
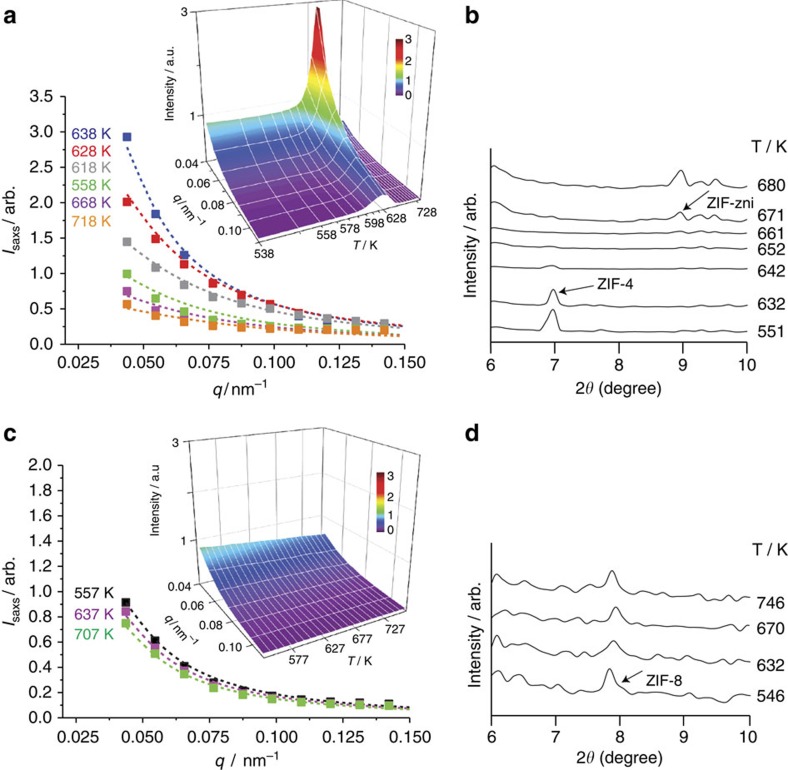
SAXS/WAXS data on ZIF-4 (top) and ZIF-8 (bottom). (**a**) *I*(*q*)_SAXS_ profiles of ZIF-4, with Lorentzian fits (Supplementary Methods) and three-dimensional plot (inset), highlighting the emergence of a peak between 618 and 663 K (Supplementary Fig. 2f). (**b**) WAXS data shows the major loss of Bragg diffraction on collapse at *ca.* 642 K. (**c**) *I*(*q*)_SAXS_ profiles with Lorentzian fits and three-dimensional plot of the SAXS results for ZIF-8. (**d**) WAXS data show the retention of crystallinity across the entire temperature range studied.

Variable temperature SAXS and WAXS measurements were performed to probe the mechanism of amorphization. The SAXS signal *I*(*q*)_SAXS_, which measures differences in local density^12^, crucially continues after the majority of Bragg diffraction disappears, the SAXS maximum extending to significantly higher temperatures (Fig. 2a,b and Supplementary Fig. 2), as found earlier for conventional zeolites^13^. In this case it supports the coexistence of LDA and HDA phases for the amorphization of a hybrid system, so-called polyamorphic phases identical in composition but different in density and entropy. In contrast to ZIF-4, the structural integrity of ZIF-8 was maintained throughout the heating process (Fig. 2c,d).

## Discussion

**Figure 3:**
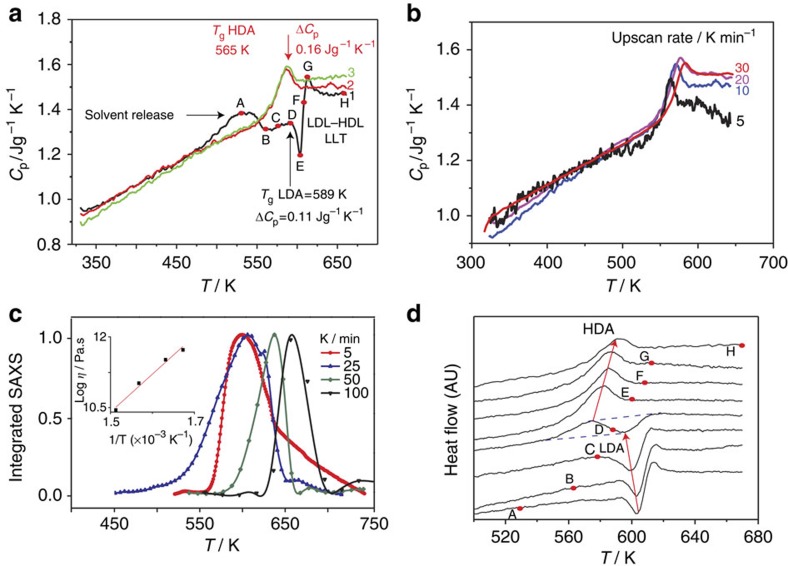
Dynamics of ZIF-4 amorphization, polyamorphic glass transitions and coexistence (**a**) Sequence of DSC up-scans on ZIF-4 at 10 K min^−1^ starting with ZIF-4 (black), showing: solvent release (A), collapse to LDL phase (D–F), followed by the LLT to HDL (F–H). The jump in the isobaric heat capacity (*C*_p_) through the LLT (E–G) is 0.33 J g^−1^ K^−1^. Δ*C*_p_ is the difference in *C*_p_ from glass to liquid at *T*_g_, being 0.11 and 0.16 J g^−1^ K^−1^ for LDA and HDA phases, respectively. The endotherms in successive scans (2–red, 3–green) relate to HDA phase. (**b**) DSC second up-scans on the same samples at different rates right after cooling, yielding *T*_g_ and *m* for HDA. (**c**) The change in integrated SAXS 
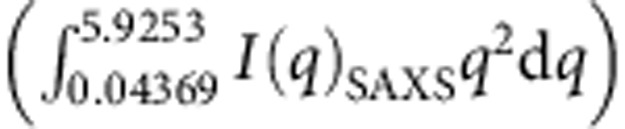
, showing the increase of the peak temperature (*T*_peak_) for different heating rates, giving *T*_g_ and *m* for the LDA phase. Inset: dependences of the Maxwell viscosity^12^ *η=G*_*∞.*_*τ*, where *G*_*∞*_ and *τ* are the adiabatic shear modulus (2 GPa)^40^ and structural relaxation time ∼1/heating rate, respectively. (**d**) DSC up-scans preheated to temperatures A(529 K), B (563 K), C(578 K), D(588 K), E(601 K), F(608 K), G(613 K), H(673 K), cooled back to room temperature, and then reheated to 673 K—all at 10 K min^−1^. Arrows indicate *T*_g_ HDA increasing and *T*_g_ LDA decreasing with increases in initial scan temperature. Temperature at 588 K reveals coexistence of LDA and HDA. With double scans (**d**), amorphization stages occur 20 K lower than for single scans (**a**).

The DSC upscan curves of ZIF-4 (Fig. 3a, Supplementary Fig. 4) display endotherms (A) from release of framework templating *N*,*N*-dimethylformamide (DMF) , which does not cause framework collapse. An exothermic feature follows (D–F), which indicates LDA *T*_g_ (Fig. 3a) (confirmed by SAXS experiments, Fig. 3c). Above this temperature, the resultant LDL converts to a HDL (Fig. 3a F–H)—corresponding to the HDA phase heated above *T*_g_ (Fig. 3d). This order–disorder transition is similar to polyamorphic transitions in inorganic zeolites^4^,^13^ and glass-forming liquids^12^. Further heating results in recrystallization of HDL to ZIF-zni^18^. When the HDL is cooled to room temperature, after completion of the liquid–liquid transition (LLT), the HDA phase forms. This is confirmed by the occurrence of the glass transition at *T*_g_=565 K during reheating of the HDA, which is significantly lower than LDA *T*_g_=589 K (Fig. 3b), as found earlier in amorphizing inorganic zeolites. At the same time, the HDA–LDA LLT (H–C Fig. 3a) is not retraced at least with the DSC cooling rates currently available.

**Figure 4:**
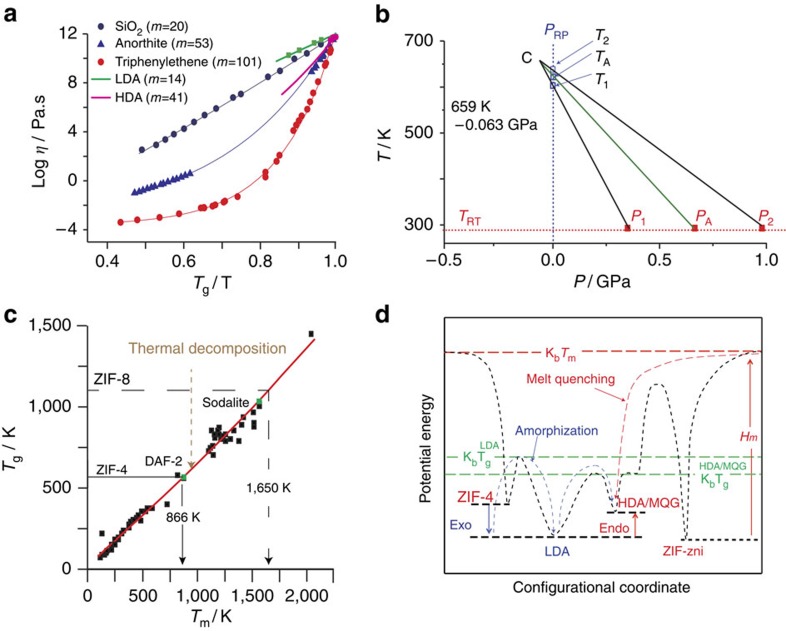
Fragilities and critical point of ZIF-4 polyamorphs, projecting *T*_m_ from *T*_g_, and PEL schematic of ZIF-4 amorphization, melting and quenching routes. (**a**) Angell plot showing the fragility of LDL and HDL ZIF-4 (Fig. 3b,c), alongside other glass-forming liquids^41^ including the silica with <20 p.p.m. hydroxyl and <60 metallic impurities. Solid lines are fits to the measured viscosity-temperature relation of the model derived in previous literature^16^,^42^. (**b**) *T*–*P* phase diagrams obtained from the limiting thermobaric amorphization parameters for ZIF-4 *P*_1_, *P*_2_, *T*_1_ and *T*_2_, which extrapolate to a critical point C at negative pressure *T*_c_ (659 K) and *P*_c_ (–0.063 GPa). *P*_A_ and *T*_A_ refer to 50% amorphization points under pressure (RP)^5^ and temperature (RT)^18^, respectively. (**c**) 2/3's Law (*T*_g_ versus *T*_m_) for different glass-forming systems^29^,^30^,^31^, including ZIF-4 and ZIF-8 compared with DAF-2 and sodalite, respectively. The thermal degradation temperature separating the locations of the two amorphized ZIFs is shown. (**d**) Schematic of the PEL^43^ for ZIF-4, informed from DSC experiments from Figs 1b and 3a. The adjacent LDA and HDA minima bear resemblance to the two states for water, different in density and topology, recently identified in modelling ST2 water^36^.

The various stages of amorphization are significantly heating rate dependent (Fig. 3b,c, Supplementary Fig. 4), from which the striking differences in fragility between HDL and LDL phases can be obtained (Fig. 4a). Remarkably, coexistence of LDA and HDA phases in the sample during amorphization is captured from double DSC scans (Fig. 3a,d), by progressively preheating to temperatures from desolvation, through collapse to the polyamorphic LDL–HDL transition (curves A–H in Fig. 3d). This can clearly be seen in curve D, where the endothermic response is followed by an exothermic one. The first relates to the *T*_g_ of the HDA phase already formed, with the second further collapse of ZIF-4 initiated at the *T*_g_ of LDA phase. The increase in the former follows the expected trend with increased pretreatment temperature, while the smaller but opposite trend in the latter suggests some increase in degrees of freedom as collapse advances. At coexistence (Fig. 3d) the 24 K difference between HDA and LDA glass transitions is reproduced. By comparison, single scanning (Fig. 3a), starting from ZIF-4 after solvent release, progresses consecutively through the respective transitions ZIF-4 to LDA (exothermic) and LDL–HDL (endothermic).

The large differences in viscosity of the glass-forming LDL and HDL phases can be quantified via Angell plots (log *η* versus *T*_g_/*T*; Fig. 4a), with respective fragilities of *m*=14 and 41 resulting from use of structural relaxation times in SAXS (Fig. 3c) and DSC experiments (Supplementary Figs 5 and 6). Arrhenius ZIF-4→LDL collapse is hence what is expected for very strong liquids (*m*=14), while HDL (*m*=41) has intermediate fragility (Fig. 4a); this is in comparison to silica which is strong, the fragile anorthite and the very fragile triphenylethene. Given the melt fragility of silica (*m*=20) (Fig. 4a), the LDL phase (*m*=14) is referred to as a super-strong liquid^8^,^12^ and controls temperature induced collapse^4^.

ZIF-4 also collapses with pressure at room temperature between 0.35 GPa (*P*_1_) and 0.98 GPa (*P*_2_) (ref. 5), equivalent to thermal amorphization at ambient pressure between 603 K (*T*_1_) and 638 K (*T*_2_) (ref. 18). In accordance with prior work on zeolite instability, a *T*–*P* phase diagram similar in form to the two-liquid model of Rapoport^4^,^24^ is shown in Fig. 4b, constructed from *P*_1_, *P*_2_, *T*_1_ and *T*_2_ (refs 4, 13). These are the pressure and temperature amorphization limits for the collapse of ZIF-4 and approximate to the spinodal limits for LDA and HDA phases. The negative d*T*/d*P* slope and the increase in entropy through the LDA–HDA transition (Fig. 1b), as discussed earlier in the context of the Clapeyron relation and the amorphization of inorganic zeolites^13^, reaffirms the low and high densities of the ZIF-4 LDA and HDA phases. Furthermore, from the LDA–HDA excursion in *C*_p_ (E–G in Fig. 3a), the entropic rise between the two phases ΔS_LDA–HDA_ (66 Jmol^−1^ K^−1^) yields through d*T*/d*P*=Δ*V*_LDA–HDA_/Δ*S*_LDA–HDA_ a shrinkage of the molar volume of ZIF-4 (337 cm^3^) ΔV_LDA–HDA_ of 10%. Following the line of enquiry in Fig. 4b, and extrapolating back the limits of the amorphization process, ZIF-4 LDA (*P*_1_→*T*_1_) and LDA–HDA (*P*_2_→*T*_2_), the LDA and HDA phases of ZIF-4 should become coexistent and identical at a critical point C at negative pressure, equivalent to what is observed in inorganic zeolites^3^,^13^, and indeed similar to that predicted for amorphous silicon^25^ and in yttria-alumina supercooled liquids^26^. Critical points are associated with a sharp increase in density fluctuations^27^, which, in Fig. 4b, will extend to ambient pressure, explaining the sharp peak in *I*(*q*)_SAXS_ (Fig. 2a). The fact that the SAXS line shapes are closely Lorentzian is also consistent with the Ornstein–Zernike model for scattering close to critical points^28^.

The chemical structures of ZIF-4, HDA and MQG phases probed by X-ray total scattering data are shown in Supplementary Fig. 11. All of the pair distribution functions (PDFs, Fig. 1c) below 6 Å contain very similar sharp features, confirming the retention in HDA and MQG of the organic ligand and zinc tetrahedral coordination environments that characterize ZIF-4 (Fig. 1a). Given the similarity of MQG and HDA PDFs, along with the reconstructive transition from HDA to ZIF-zni^18^, we infer that some degree of Zn–N bond reconstruction occurs during amorphization and melting. Intriguingly, macroscopic flow of the melt into a non-porous glass (Brunauer-Emmett-Teller (BET) surface area of <5 m^2^ g^−1^) can be seen in scanning electron microscopy (SEM) and optical images (Fig. 1c, Supplementary Figs 7 and 8). The light brown color persists, even when oxygen is excluded from the reaction (Supplementary Fig. 9). At the same time ^1^H NMR data recorded on digested samples confirm that imidazolate ligands (Fig. 1a) remain largely intact (Supplementary Fig. 10).

The LDA glass transition temperature *T*_g_ (589 K) is extremely close to 2/3 *T*_m_ for ZIF=4 (866 K), and therefore complies with the empirical law found for many glasses^29^ (Fig. 4c). Interestingly, another MOF of Zn(Im)_2_ composition (ZIF-3), possesses the ‘dft' zeolitic topology and undergoes identical amorphization and recrystallization to ZIF-4 (ref. 7). The inorganic cobalt phosphate framework DAF-2 (also adopting the ‘dft' topology) is observed to melt at 873 K (ref. 22), indicating that frameworks with similar network topologies may exhibit similar melting behaviour, which may in turn be driven by collective THz modes^6^. In contrast, for Debye solids like dense minerals and metals, melting is activated by nearest neighbour *r*_NN_ vibrations when √(Δ*r*^2^_NN_)/*r*_NN_≥0.1—Lindemann's Law^21^.

The 2/3's Law, already well-established for molecular and network glass formers^30^,^31^ and extended in Fig. 4c to include oxide glasses, enables *T*_m_ to be projected from the LDA *T*_g_, where zeolites and MOFs collapse. In particular, despite ZIF-8 undergoing thermal decomposition before melting (Fig. 1b), a ‘hypothetical' *T*_g_ of 1,100 K, can be calculated from the relationship *P*_A_Δ*V*_A_≈3RT_g_ (refs 4, 7, 23), where *P*_A_ is the amorphization collapse pressure and Δ*V*_A_ the collapsed volume. Using Fig. 4c this projects a ‘virtual' *T*_m_ for ZIF-8 at 1,650 K. The temperature, which in practice is not achieved before decomposition of the hybrid framework, lies close to *T*_m_ of the inorganic analogue (sodalite), which melts at 1,557 K. We postulate that, by this methodology, comparison of MOFs with their inorganic analogues should reveal candidates with achievable melting points and which could therefore form hybrid melt-quenched glasses like ZIF-4.

Fundamental to current understanding of the 2/3's Law^30^,^31^ is the kinetic fragility of the melt *m* and its association with the thermodynamic variables: heat of fusion, *H*_m_ and the jump of the isobaric heat capacity (*C*_p_) from the glass at *T*_g_ to its liquid state above *T*_g_, Δ*C*_p_(*T*_g_) viz, 
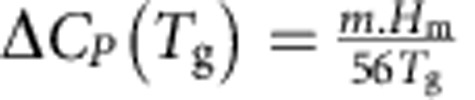
. Recognizing the relationship between melt fragility and Poisson's ratio for the glass^32^, we have adapted this empirical relationship and recovered Δ*C*_p_ values (Fig. 3a) measured for LDL and HDL phases (Supplementary Methods, Supplementary Table 1). In addition, considering the various enthalpy changes occurring during amorphization, crystallization and melting (Fig. 1, Supplementary Fig. 3), a schematic PEL incorporating ZIF-4, LDA, HDA and MQG is plotted in Fig. 4d. It shows strong similarity with the behaviour of zeolite-A and its polyamorphs (Fig. 4d)^13^. It also illustrates the likely common location of HDA and MQG in configuration space, reached by amorphization and by melt quenching, respectively.

Finally, we consider the fact that, for these current experiments, cooling of the HDL ZIF phase does not result in formation of the LDL phase, encountered on heating (Fig. 3a). In terms of potential energy, the LDA ZIF phase shares similarities with the ultrastable glass obtained by molecule-by-molecule coating^15^. This also has a *T*_g_ greater than that of its ‘normal' HDA glass counterpart. On reheating the ultrastable glass is transformed into the normal glass state, though does not convert back to the ultrastable state within the cooling rates available using these techniques. This is also the same scenario observed in aged amber^33^. By contrast, the LDA ZIF-4 phase reported here coexists with the HDA phase (Fig. 3d). When ZIF-4 has fully released its solvent, its structural arrangement becomes looser, but still remains ordered with an unchanged potential energy. With further heating the structure of solvent-free ZIF-4 relaxes towards a more stable state and lower enthalpy level, enthalpy being released with formation of the LDL phase (Figs 1b and 4d). This behaviour is also common to anhydrous zeolites^4^,^13^, whose enthalpies all exceed those of conventional oxide glasses with the same composition^34^, reflecting the metastable nature of zeolitic crystals. When the temperature subsequently rises above *T*_g_, the LDL phase spontaneously transforms into the HDL ZIF phase. Beyond that, the HDL phase is finally turned to a more stable ZIF-zni crystal phase.

This apparent irreversibility for the LLT described here for the amorphization of ZIF-4 is in contrast to the reversibility reported in *ab initio* Molecular Dynamics volume versus pressure calculations on zeolites^35^. With increasing pressure, zeolite-LDA followed by LDA–HDA first order transitions could also be retraced (albeit with some hysteresis) by reducing pressure, eventually including tension^35^. Some evidence for phase transition reversibility was found experimentally during the initial zeolite collapsing process^13^. Elsewhere, experiments on yttria-alumina melts, where the LDL and HDL phases formed in levitated liquid drops, were observed to fluctuate back and forth at the LLT^26^. Similar behaviour is confirmed both by the recent modelling of ST2 water^36^ and experimental work on mannitol^37^.

The apparent irreversibility of the LLT in ZIF-4 on current experimental timescales may lie kinetically in the inherent structural differences between hybrid and inorganic systems, in particular the comparative rigidity of the inter-tetrahedral bridging unit^2^. Compared with oxide melts and zeolites, for example, the floppy bridging oxygen is replaced by the rigid imidazolate bridge in ZIF-4 (Fig. 1a). This will influence differences in conformational changes involved in the order–disorder LDA–HDA transition that determines the HDA topology, and may not be kinetically symmetric. So, while the entropy of the HDA phase lies comparatively close to that of the low-density state (Figs 1b and 3a), and both phases can coexist on heating (Fig. 3d), the difference is that for systems like water^36^ and yttria-alumina melts^26^, that are readily reversible, these appear to fluctuate freely between separate free energy basins facilitated by pivotal inter-polyhedral bridges. In particular, where the LDL to HDL transition in ZIF-4 is thermodynamically controlled, the reverse process appears to be kinetically controlled, the dynamics being out of range using current experimental methods.

Comparisons between amorphization and melting conditions of MOFs and inorganics may provide further routes to more functional ‘perfect' glasses, HDA and MQG phases. Furthermore, the *in situ* hybrid liquid formation discovered here opens up possibilities for liquid casting and shaping MOFs into a variety of different solid forms, promising to be an extremely exciting step forward in producing chemically functionalizable hybrid glass materials.

## Methods

### Synthesis

1.2 g of Zn(NO_3_)_2_·6H_2_O and 0.9 g of imidazole were dissolved in 90 ml of DMF and transferred into a 100 ml screw jar. The jar was tightly sealed and heated to 100 °C for 72 h in an oven. After cooling to room temperature colourless block-shaped crystals were filtered off and first washed three times with ∼30 ml pure DMF and then three times with ∼30 ml CH_2_Cl_2_. The HDA and quenched ZIF-zni used for the PDF experiments were formed by heating ZIF-4 to 573 and 865 K under an argon atmosphere using a ramp rate of 5 K min^−1^.

To investigate the effect of oxygen on the process, a 1 mm diameter quartz tube was loaded with a sample of crystalline ZIF-4, and sealed under vacuum. The capillary was then heated in a tube furnace under an argon flow, at a rate of 5 °C min^−1^, to 865 K. The final melt-quenched glass was not observed to differ from that attained in other experiments.

For SAXS and WAXS measurements, the crystals were gently stirred in 100 ml fresh CH_2_Cl_2_ overnight. Afterwards the solid material was filtered off, washed again three times with ∼30 ml fresh CH_2_Cl_2_ and dried in vacuo at 130 °C, using a vacuum oven to yield activated guest-free ZIF-4.

ZIF-8 was purchased from Sigma Aldrich and evacuated by heating at 100 °C for 3 h.

### Measurements

Room temperature X-ray total scattering data were collected at the I15 beamline at Diamond Light Source, UK, at a wavelength of *λ*=0.1722 Å. Finely powdered samples of ZIF-4, HDA and MQG samples were carefully loaded into 1.0 mm diameter fused silica capillaries, and data from an empty instrument and capillary were also collected for background subtraction. Data were collected between ∼0.5<Q<∼22 Å^−1^. Corrections for background, multiple scattering, container scattering, Compton scattering and absorption were applied. The normalized reciprocal space data (Supplementary Fig S1) were then converted to the PDFs using Fourier transform.

Temperature dependent *in situ* SAXS and WAXS measurements were performed on Beamline I22 at the Diamond Light Source synchrotron in the Rutherford Appleton Laboratory (Didcot, Oxfordshire, UK). Detector calibrations were carried out using NBS (National Bureau of Standards) silicon and silver behenate standards on the HOTWAXS 1D WAXS, and RAPID 2D SAXS, detectors respectively^38^,^39^.

Normalization for beam intensity and sample thickness and/or density variation was carried out using a diode embedded in the beamstop. Scattering data were recorded at a wavelength of 1 Å for angular range of up to 1° for SAXS and over a 2*θ* range of 5°–40° for WAXS. Powdered samples of ZIF-4 and ZIF-8 were loaded in glass capillaries and inserted horizontally through the Linkam furnace, which was positioned across the synchrotron radiation source. The Linkam furnace used was calibrated, finding the relationship *T*_True_=0.95 T (set point, C)+65. Raw data for temperature scanned SAXS ZIF-4 and ZIF-8 are shown in Supplementary Fig. 2. SAXS profiles were fitted to Lorentzian line shapes, 
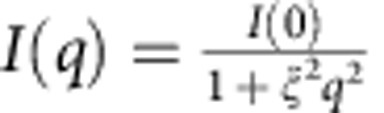
, with the correlation length ξ increasing from ∼220 to ∼840 Å from the edge to the peak (Fig. 2a). Additional measurements accompanied by a detailed analysis will be given in a separate publication.

The apparent isobaric heat capacity (*C*_p_) of each sample was measured using a Netzsch STA 449C DSC. The samples were placed in a platinum crucible situated on a sample holder of the DSC at room temperature and subjected to varying numbers of up- and down-scans, depending on the purpose of the measurements. After natural cooling to room temperature, the subsequent up-scans were performed using the same procedure as for the first.

Powder X-ray diffraction measurements on evacuated, guest-free ZIF-4 were recorded on a well ground sample with a Bruker D8 Advance powder diffractometer using Cu*Kα* radiation (*λ*=1.5418 Å) and a LynxEye position sensitive detector in Bragg–Brentano (*θ*−*θ*) geometry at room temperature. Pawley fit shown in Supplementary Fig. 11.

Microanalysis was performed at the Department of Chemistry, University of Cambridge as a technical service.

*ZIF-4 evacuated*. Calculated (based on Zn(C_3_H_3_N_2_)_2_ composition): C 36.18%, H 3.02%, N 28.14%. Found: C 36.22%, H 2.98%, N 28.09%

*MOF glass*. Calculated (based on Zn(C_3_H_3_N_2_)_2_ composition): C 36.18%, H 3.02%, N 28.14%. Found: C 35.64%, H 2.90%, N 26.46%

SEM images (Supplementary Fig. 7) were taken with an FEI Nova NanSEM (field emission gun). Specimens for SEM analysis were prepared by dispersing fragments of the ZIF-4 melt-quenched glass on conductive carbon tabs for topographic contrast imaging. Optical images of ZIF-4, ZIF-zni and recovered melt-quenched glass are shown in Supplementary Fig. 8.

Liquid phase ^1^H NMR spectra (Supplementary Fig. 10) of digested samples (DCl/D_2_O/DMSO-*d*_6_) of evacuated ZIF-4 and the ZIF-4 glass (∼5–10 mg) were recorded on a Bruker Avance DPX-250 spectrometer at 293 K in a mixture of DCl (35%)/D_2_O (0.1 ml) and DMSO-*d*_6_ (0.5 ml). Chemical shifts are given relative to tetramethylsilane and were referenced to the residual protio-solvent signals of DMSO-*d*_6_. The spectra were processed with the MestreNova Suite.

Nitrogen adsorption Brunauer-Emmett-Teller (BET) measurements were carried out at 77 K using a Micromeritics ASAP 2020 instrument.

## Additional information

**How to cite this article:** Bennett, T. D. *et al.* Hybrid glasses from strong and fragile metal-organic framework liquids. *Nat. Commun.* 6:8079 doi: 10.1038/ncomms9079 (2012).

## Supplementary Material

Supplementary Information Supplementary Figures 1-12, Supplementary Table 1, Supplementary Methods and Supplementary References
