# Supplementary material for: Hybrid glasses from strong and fragile metal-organic framework liquids
Source: Nat Commun. 2015 Aug 28;6:8079. doi: 10.1038/ncomms9079 (PMC4560802; doi:10.1038/ncomms9079)
Supplement: Supplementary Information — Supplementary Figures 1-12, Supplementary Table 1, Supplementary Methods and Supplementary References [file ncomms9079-s1.pdf]

Supplementary Figures

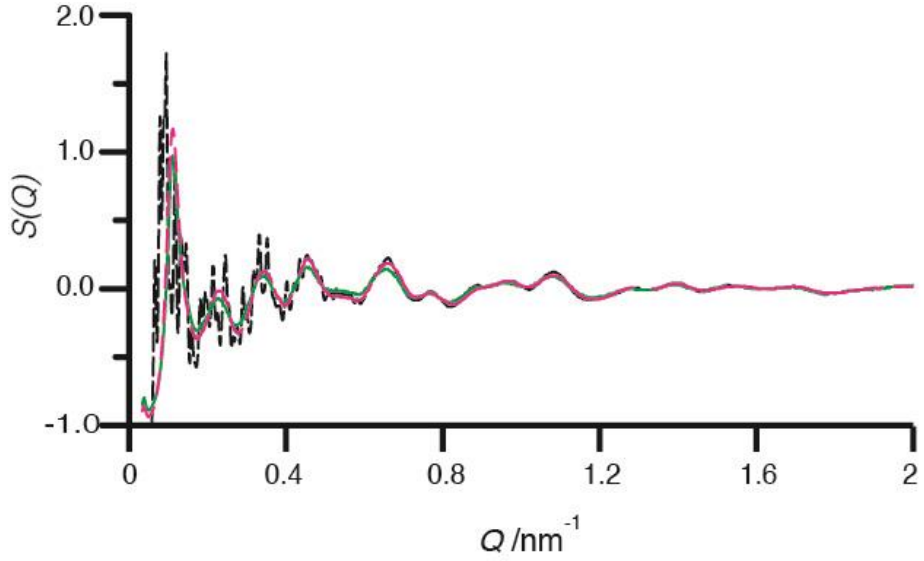

Supplementary Figure 1. X- ray total scattering data measured for samples. MQG (green), HDA (broken pink) and crystalline ZIF-4 (broken black) samples. The absence of Bragg peaks for the MQG and HDA samples confirms their amorphous nature. Corrections for background, multiple scattering, container scattering, Compton scattering and absorption were applied using the GudrunX program.[1]

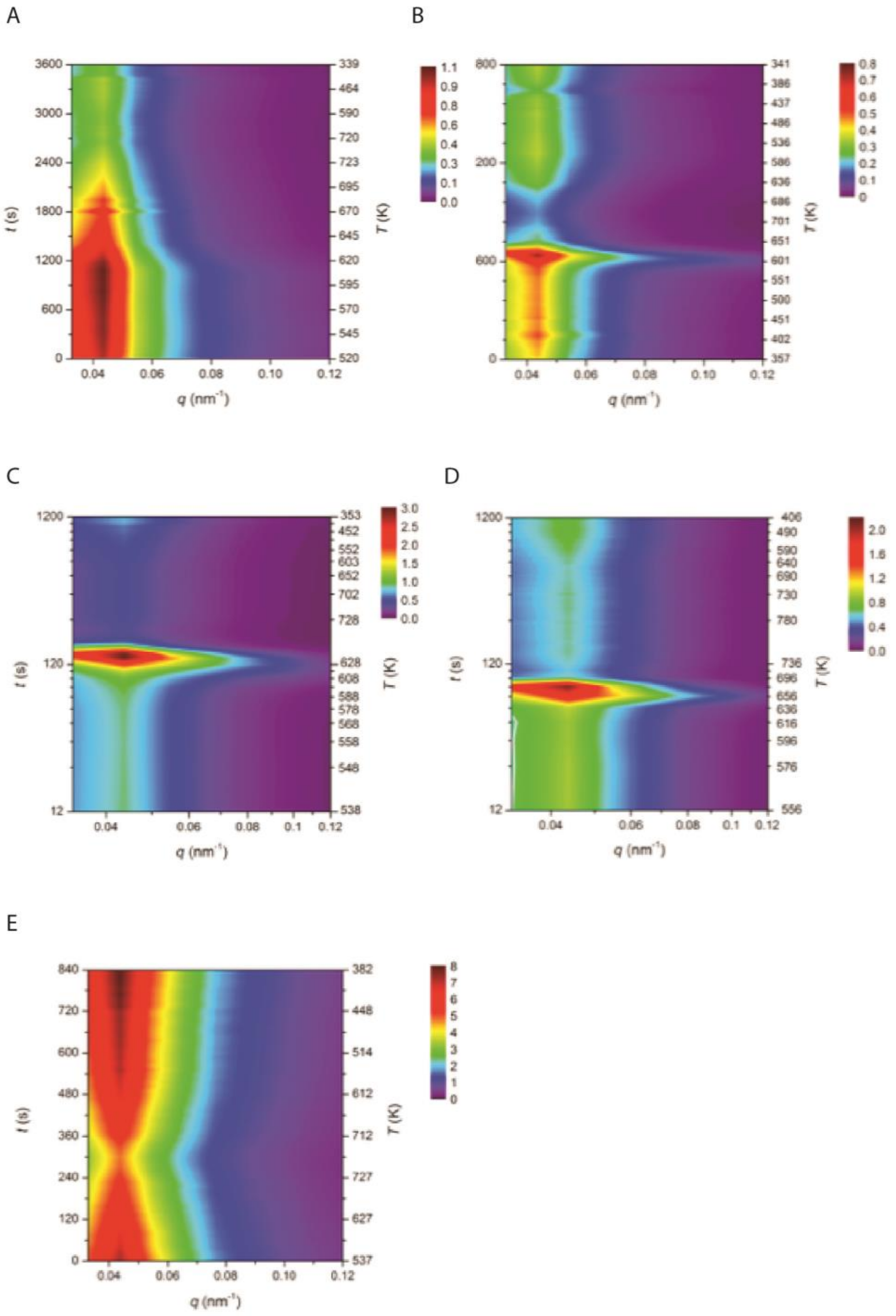

Supplementary Figure 2. Raw SAXS data on amorphization. SAXS data collected *in-situ* for ZIF-4 using heating rates of (a) 5 K min^{-1} (b) 25 K min^{-1}. (c) 50 K min^{-1} and (d) 100 K min^{-1}. Data also included for ZIF-8 at 25 K min^{-1} (e).

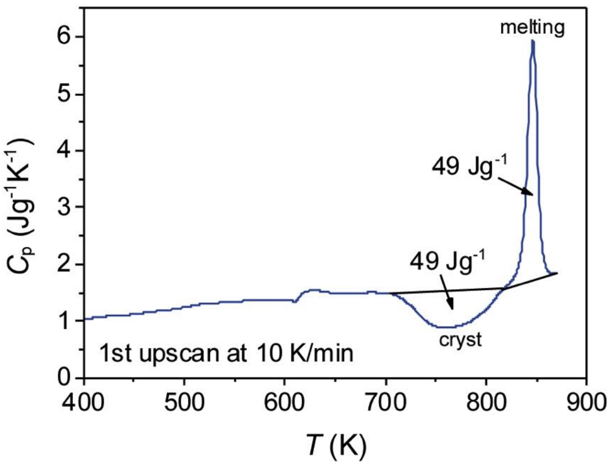

Supplementary Figure 3. DSC measurement on ZIF-4 performed at a rate of 10 K min^{-1}. The exothermic crystallization of the HDA phase at *ca*. 770 K is of the same enthalpy as the endothermic melting of ZIF-zni to form a hybrid liquid.

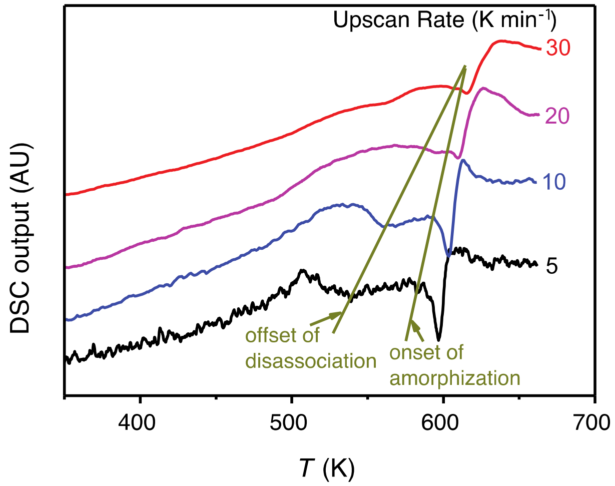

Supplementary Figure 4. Dynamics of ZIF-4. a) DSC first upscans on ZIF-4 samples at different heating rates. The two straight lines refer to both the offset of the solvent disassociation and the onset of the amorphisation of ZIF-4, respectively.

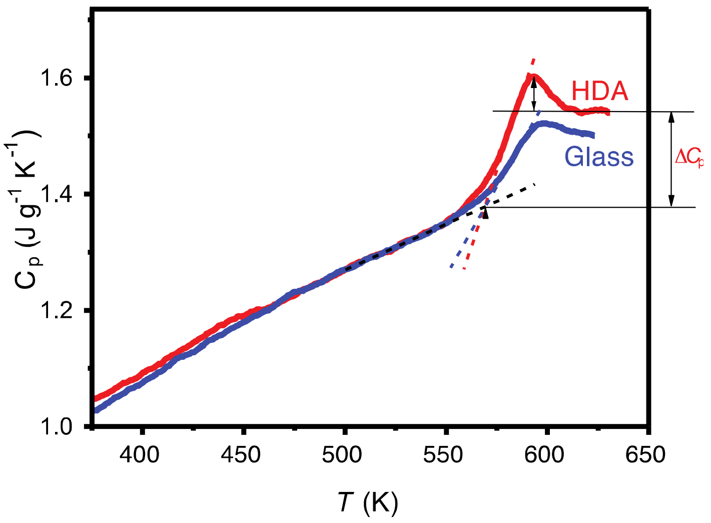

Supplementary Figure 5. Comparison of glass transitions. The heat capacity (C_p) curves obtained at the rate of 20 K min^{-1} for the HDA phase of ZIF-4 and the glass obtained by quenching the ZIF-4 melt; both exhibiting the same T_g (565 K).

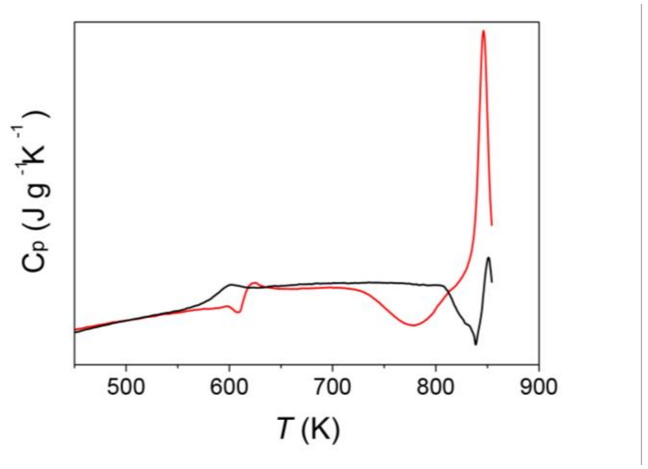

Supplementary Figure 5b. Comparison of the recrystallization behavior. ZIF-4 (red) and the MQG (black), the latter formed by cooling molten ZIF-zni at 40 K min^{-1}. Recrystallization to ZIF-zni is observed in each case. Heating rate 20 K min^{-1}.

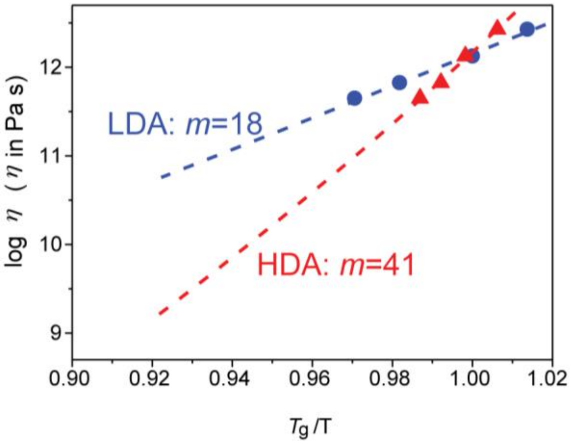

Supplementary Figure 6. Fragilities of LDA and HDA. The fragility index *m* is determined from the dependence of fictive temperature (T_f) on the heating rate (q_h) by using the DSC method (Figs. 3a and b). Because of confusion between ZIF-4 solvent release endotherm and the LDA T_g (Supplementary Figure 7), together with the limit of (equ. 1) to $m \geq 15$, fragility of the LDL phase was estimated to be m=18±2. Following earlier procedures used for obtaining T_g and *m* from the collapse of inorganic zeolites (5,14), more accurate values for LDA were obtained from the dependences of the reciprocal heating rate ($1/q_h$) on the T_g-scaled peak temperature T_{peak} (Fig. 3c). These yielded m=14 and T_g = 589 K, the LDA fragility being ~1/3 that of HDA. Angell plots contrast the respective fragile and ultra-strong viscosity relations of HDA and LDA in Fig. 4a.

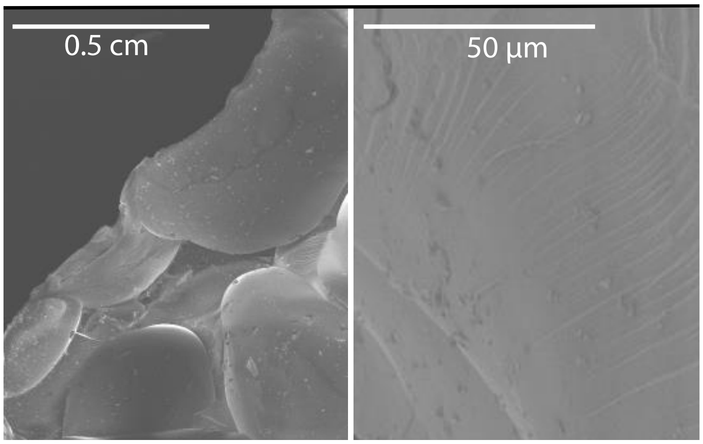

Supplementary Figure 7. Scanning electron micrograph images of the MQG. Thermal stress-induced striations are evident.

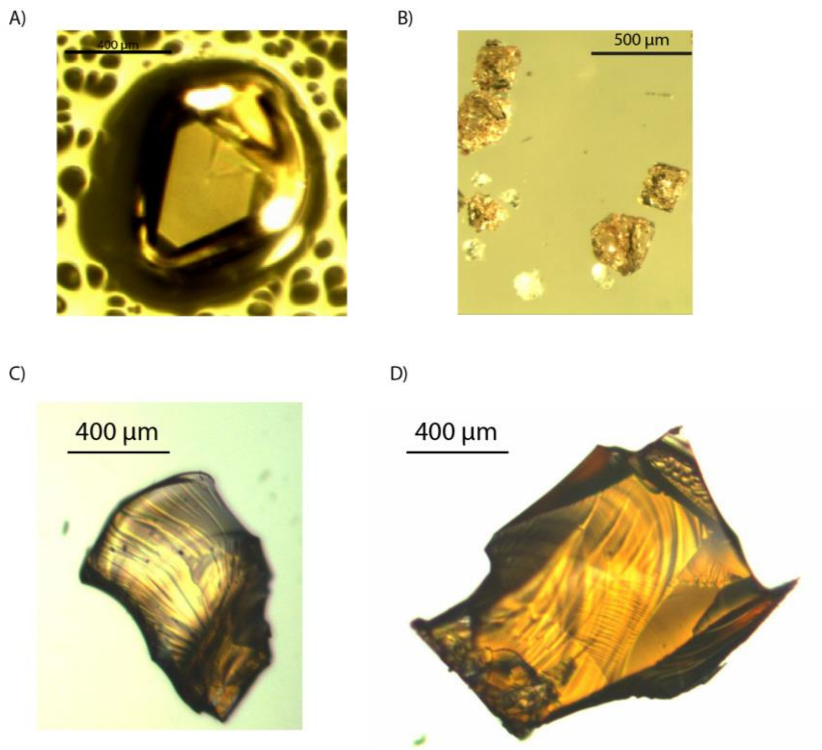

Supplementary Figure 8. Optical images of the glasses. (a) ZIF-4, (b) ZIF-zni and (c,d) the MQG

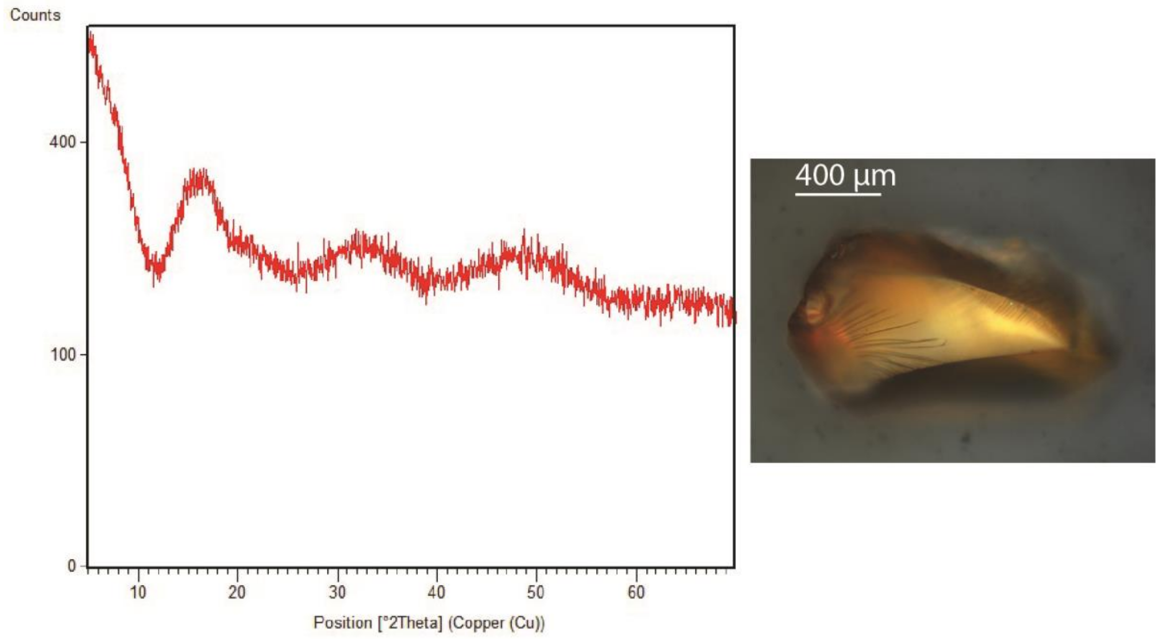

Supplementary Figure 9. Oxygen exclusion. Powder X-ray diffraction pattern and optical image of the MQG formed when oxygen is excluded.

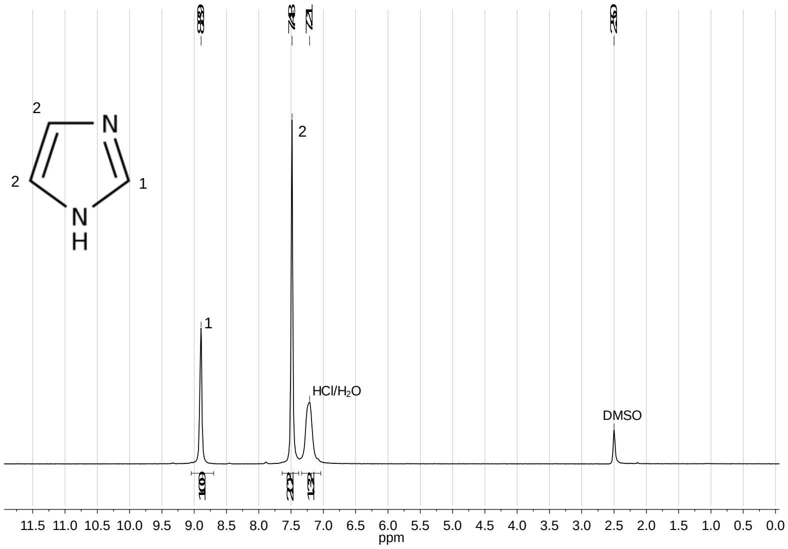

Supplementary Figure 10a. **^{1}H NMR spectrum of ZIF-4.** A digested sample of evacuated ZIF-4 in $DCl/D_2O/DMSO$-d_6.

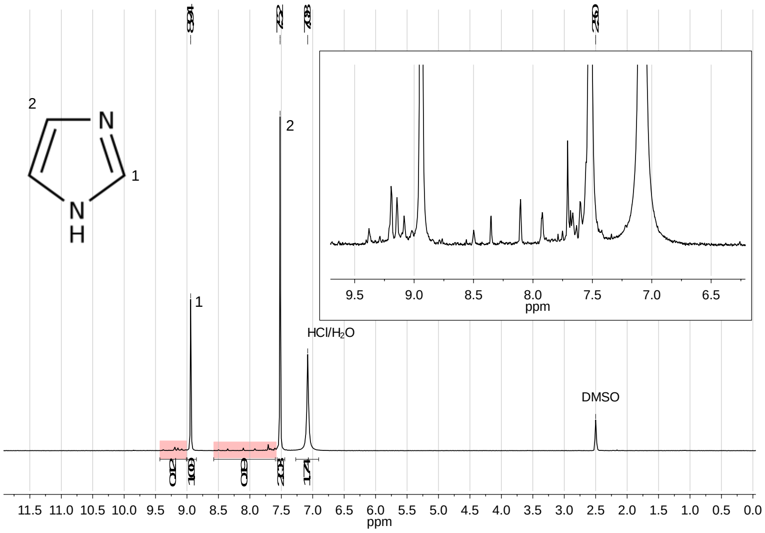

Supplementary Figure 10b. **^{1}H NMR spectrum of ZIF-4 glass.** A digested sample of the MQG in $DCl/D_2O/DMSO$-d_6. The inset shows a closer view on the various impurity peaks of low intensity (also marked in light red in the main section). The integrated intensity of the impurity peaks suggests that less than 10% of the imidazolates (partially) decompose during melting.

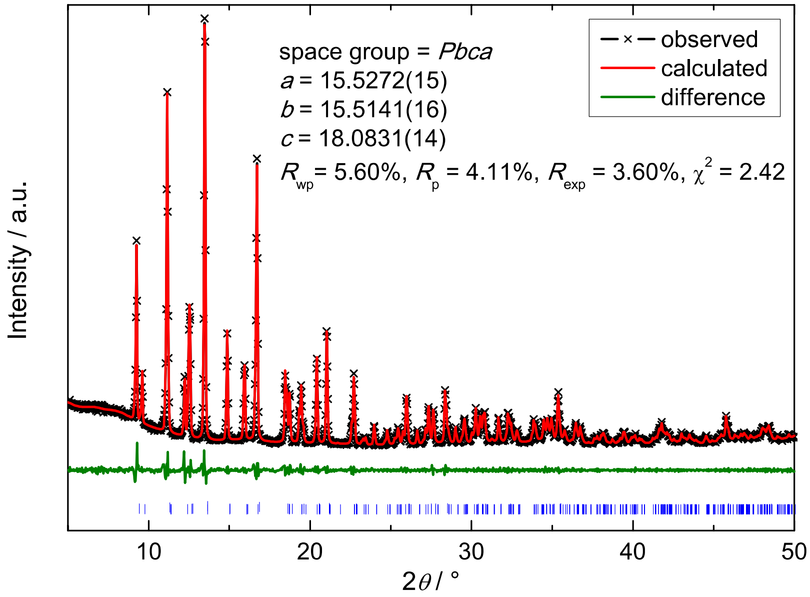

Supplementary Figure 11. Pawley fit[2] to the diffraction pattern of ZIF-4. The black crosses, red lines, and green lines represent the experimental, calculated, and difference profiles, respectively. The blue tick marks indicate the positions of allowed Bragg reflections in the space group *Pbca*. Pawley fitting was performed with the TOPAS program package (academic version). The crystallographic parameters of the crystal structure of as-synthesized ZIF-4 from Park *et al.* were used as a starting model [3].

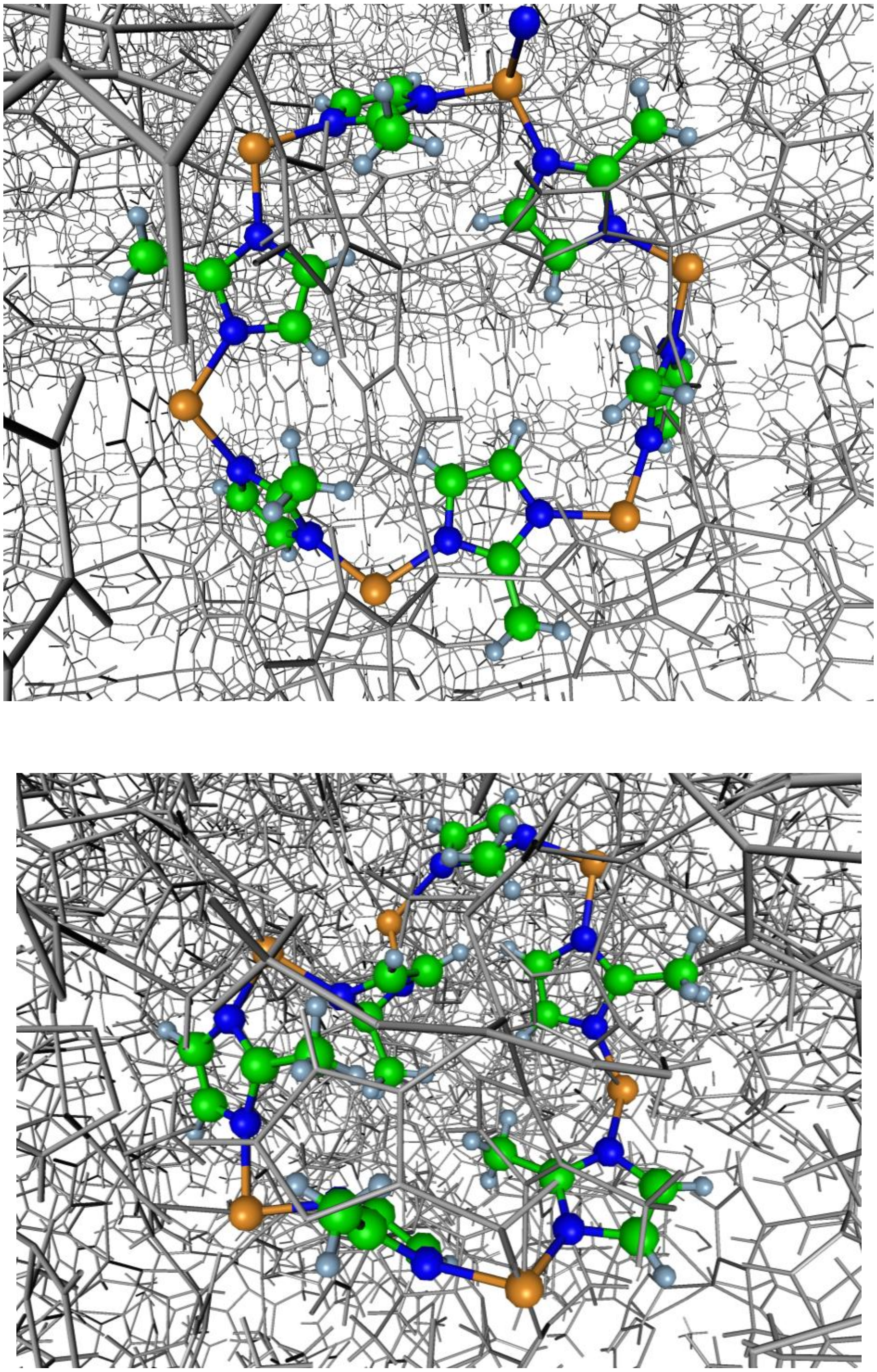

Supplementary Figure 12. Molecular graphics. Pressure induced amorphization using DLPOLY classic [9]. ZIF-8 at ambient pressure (top) and after increasing the pressure to 1.2 GPa (bottom). 6-fold ring with imidazolate linkages are included in each case.

	$\Delta C_p\,(T_g)$	T_g (K)	*m* eg.3	*m* eq. 4	*m* Fig.4a
ZIF-4 LDL	0.11	589	74.1	14	14
ZIF-4 HDL	0.165	565	106	38	41

Supplementary Table 1. Endothermic step $\Delta C_p(T_g)$, and fragilities calculated from eqn. 3 which overestimates those measured from DSC, and eqn. 4 which is in much better agreement.

Supplementary Methods

To determine the heat capacity (C_p) of the samples, both the baseline (blank) and the reference sample (sapphire) were measured. In order to confirm reproducibility, the measurements for some samples were repeated to check for drift in the baseline.

The fictive temperatures (T_f) of samples were determined from the second up-scan curve of heat capacity (C_p) obtained at a heating rate equal to the prior down-scan rate using DSC. T_f is assigned as the temperature at the cross point between the extrapolated linear fits to the C_p of the glass and to the rapidly rising C_p. The standard glass transition temperature (T_g) was determined from the second up-scan curve obtained at the rate of 10 K min^{-1} (Fig. 3b). The ZIF-zni crystallization exotherm and melting endotherm on fusion were obtained as shown in Supplementary Figure 3 and described in the text (Fig. 1b).

Melt fragility *m* for HDL was determined from *T*g values using the MYEGA equation :

$$\log \eta = \log \eta_\infty + (12 - \log \eta_\infty)\frac{T_g}{T}\exp\left[\left(\frac{m}{12 - \log \eta_\infty} - 1\right)\left(\frac{T_g}{T} - 1\right)\right] \qquad (1)$$

η_∞, the viscosity at 1/T=0, was set at 10^{-5} Pas, with the viscosity at the glass transition $\eta(T\mathrm{g})$ set to 10^{12} Pas (Fig. 4a), yielding m=41±2 and T_g = 565 K. The melt-quenched glass had virtually identical *m* and T_g values.

The ^{1}H NMR spectrum of digested ZIF-4 only shows signals for imidazolium (at 8.89 and 7.48 ppm) and the two signals for the utilized solvent mixture (at 7.21 ppm for HCl/water and at 2.50 ppm for DMSO). The ^{1}H NMR spectrum of the digested ZIF-4 glass basically shows the same signals, however, the peaks of imidazolium and

HCl/water are slightly shifted, which is ascribed to their pH sensitivity. Additionally, a number of small signals in the aromatic region at 9.4-9.0 ppm and 8.6-7.6 ppm are present (see inset of Fig. 10b). We ascribe these signals to a partial decomposition of the imidazolate linker upon amorphisation/melting. However, the integrated intensity of the impurity peaks suggests that only about 10% of the ligands undergo thermal decomposition.

Elemental analysis was performed at the Department of Chemistry, University of Cambridge. Elemental Analysis on the MQG.

ZIF-4 Evacuated:

Calculated (based on $Zn(C_3H_3N_2)_2$ composition): C 36.18 %, H 3.02 %, N 28.14 %
Found: C 36.22 %, H 2.98 %, N 28.09 %

MQG

Calculated (based on $Zn(C_3H_3N_2)_2$ composition): C 36.18 %, H 3.02 %, N 28.14 %
Found: C 35.64 %, H 2.90 %, N 26.46 %

Estimating T_g and the virtual melting point T_m for ZIF-8

The following empirical relationship was established from the collapse dynamics of of zeolite A and zeolite Y [3] between the thermally induced amorphization temperature T_A that occurs at the LDA T_g and the pressure induced amorphization pressure P_A viz.,

$$P_{A}.\Delta V_A \approx 3RT_g \qquad (2)$$

where ΔV_A is the collapse volume [3].

By comparing ZIF-4 and ZIF-8,

$$T_g\,(\text{ZIF-8}) \approx T_g\,(\text{ZIF-4}).(\Delta V_A(\text{ZIF-8})/\Delta V_A(\text{ZIF-4})).(P_A(\text{ZIF-8})/P_A(\text{ZIF-4})). \qquad (3)$$

Taking T_g (ZIF-4)=589 K (Fig. 3a), (ΔV_A(ZIF-8)/ΔV_A(ZIF-4) to be proportionate to the ratio of the respective Solvent Accessible Volumes, SAV(ZIF-8)/SAV(ZIF-4)=2471/1490=1.66, and the amorphization pressures from the literature, viz. P_A(ZIF-8)=0.75 GPa [22] and P_A(ZIF-4)=0.665 GPa [9], gives T_g (ZIF-8)≈1102 K. From Fig. 4c this projects a virtual melting point for ZIF-8 of 1650 K.

Fragility from 2/3's Law

Following [4,5] the melt fragility m for molecular liquids is empirically related to $\Delta C_p(T_g)$ (Fig. 1e, Fig. 3a, Supplementary Figure 7), by

$$m = \frac{56.T_g.\Delta C_p(T_g)}{H_m} \qquad (4)$$

where H_m is the heat of fusion (49 Jg^{-1} Supplementary Figure 3). Fragility values for LDL and HDL calculated from Table S1 are also listed and over-estimate those determined experimentally (Fig. 4a). Among molecular liquids, Wang *et al* identified similar outliers, such as for Se and PPP, which they attributed to changes in topology/connectivity[4]. Since increases in connectivity across glassy materials is inversely related to Poisson's ratio $\upsilon = [\frac{3(B/G)-2}{6(B/G)+2}]$ υ = [6], this might be used to correct overestimates of m, as melt fragility and B/G are empirically related.

$$m = \frac{56. T_g. \Delta C_p(T_g). \upsilon}{H_m} \quad (5)$$

Agreement between fragility determined from eqn. 4 and measured values is much improved.

DLPOLY classic [7] was used to perform the MD simulations (Fig. 1a). The potential model was constructed from the reference[8] including bonded and non-bonded terms. The bonded parts contain stretching, bending, proper and improper potentials, while the non-bonded parts have Lennard-Jones and Coulombic potentials.

In order to achieve pressure induced amorphization of ZIF-8, a cubic super cell with 7452 atoms was studied. Simulations were performed in the constant temperature/pressure (NPT) ensemble throughout. The temperature (275K) was controlled using berendsen thermostats with a relaxation constant of 1ps. Constant pressures varied from 1atm to 1.2GPa were maintained by applying isotropic barostats with the same relaxation constant as thermostats. The final structure of amorphous ZIF8 was obtained after the compressing of 1.2GPa for 10ps.

Supplementary References:

1 Soper, A. K. GudrunN and GudrunX: Programs for Correcting Raw Neutron and X-ray Diffraction Data to Differential Scattering Cross Section. *Tech. Rep. RAL-TR-2011-013* (2011).

2 Pawley, G. S. Unit-Cell Refinement from Powder Diffraction Scans. *J. Appl. Cryst.* **14**, 357-361 (1981).

3 Park, K. S. *et al.* Exceptional chemical and thermal stability of zeolitic imidazolate frameworks. *Proc. Natl. Acad. Sci. U S A* **103**, 10186-10191 (2006).

4 Wang, L. M., Angell, C. A. & Richert, R. Fragility and thermodynamics in nonpolymeric glass-forming liquids. *J. Chem. Phys.* **125** (2006).

5 Stevenson, J. D. & Wolynes, P. G. Thermodynamic-kinetic correlations in supercooled liquids: A critical survey of experimental data and predictions of the random first-order transition theory of glasses. *J. Phys. Chem. B* **109**, 15093-15097 (2005).

6 Greaves, G. N., Greer, A. L., Lakes, R. S. & Rouxel, T. Poisson's ratio and modern materials. *Nat. Mater*. **10**, 823-837 (2011).

7 Todorov, I. T., Smith, W., Trachenko K., and & Dove, M. Dl_poly_3: new dimensions in molecular dynamics simulations via massive parallelism. *J. Mat. Chem.* **16**, 1911-1918 (2006).

8 Hu, Z., Zhang, L., Jiang, J. Development of a force field for zeolitic imidazolate framework-8 with structural flexibility. *J. Chem. Phys.* **136**, 244703 (2012).
